# In vitro and in vivo anticancer effect of pH-responsive paclitaxel-loaded niosomes

**DOI:** 10.1007/s10856-021-06623-6

**Published:** 2021-12-04

**Authors:** Mahmood Barani, Mohammad Reza Hajinezhad, Saman Sargazi, Abbas Rahdar, Sheida Shahraki, Azadeh Lohrasbi-Nejad, Francesco Baino

**Affiliations:** 1 Medical Mycology and Bacteriology Research Center, Kerman University of Medical Sciences, Kerman, 7616913555 Iran; 2 Basic Veterinary Science Department, Veterinary Faculty, University of Zabol, Zabol, 98613-35856 Iran; 3 Cellular and Molecular Research Center, Research Institute of Cellular and Molecular Sciences in Infectious Diseases, Zahedan, 9816743463 Iran; 4 Department of Physics, University of Zabol, Zabol, 98613-35856 Iran; 5 Department of Agricultural Biotechnology, Shahid Bahonar University of Kerman, Kerman, Iran; 6 Institute of Materials Physics and Engineering, Department of Applied Science and Technology, Politecnico di Torino, Torino, Italy

## Abstract

In this study, paclitaxel (PTX)-loaded pH-responsive niosomes modified with ergosterol were developed. This new formulation was characterized in terms of size, morphology, encapsulation efficiency (EE), and in vitro release at pH 5.2 and 7.4. The in vitro efficacy of free PTX and niosome/PTX was assessed using MCF7, Hela, and HUVEC cell lines. In order to evaluate the in vivo efficacy of niosomal PTX in rats as compared to free PTX, the animals were intraperitoneally administered with 2.5 mg/kg and 5 mg/kg niosomal PTX for two weeks. Results showed that the pH-responsive niosomes had a nanometric size, spherical morphology, 77% EE, and pH-responsive release in pH 5.2 and 7.4. Compared with free PTX, we found markedly lower IC50s when cancer cells were treated for 48 h with niosomal PTX, which also showed high efficacy against human cancers derived from cervix and breast tumors. Moreover, niosomal PTX induced evident morphological changes in these cell lines. In vivo administration of free PTX at the dose of 2.5 mg/kg significantly increased serum biochemical parameters and liver lipid peroxidation in rats compared to the control rats. The situation was different when niosomal PTX was administered to the rats: the 5 mg/kg dosage of niosomal PTX significantly increased serum biochemical parameters, but the group treated with the 2.5 mg/kg dose of niosomal PTX showed fewer toxic effects than the group treated with free PTX at the same dosage. Overall, our results provide proof of concept for encapsulating PTX in niosomal formulation to enhance its therapeutic efficacy.

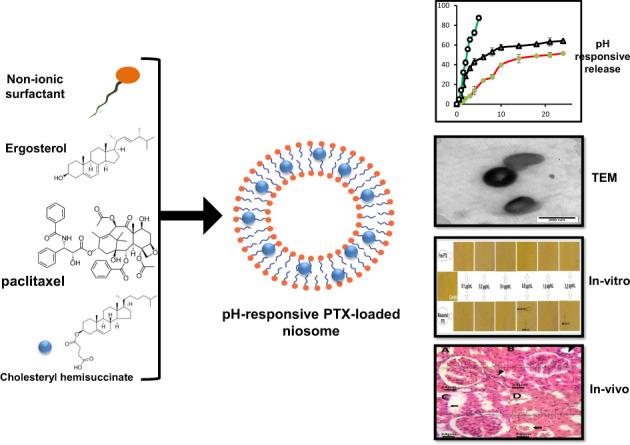

## Introduction

The active ingredient, dosage type, condition of the patient, and chemotherapy regimen all play a role in the effectiveness of cancer treatment. Therefore, improving the formulation of existing chemotherapy pharmaceutics is as necessary as inventing new anticancer drugs. In this regard, nanomedicine is being used to develop better drug forms for cancer treatment, raising new hopes for more effective therapies [1, 2]. Paclitaxel (PTX) is an anticancer drug that is beneficial against various cancers, including breast, lung, and ovarian carcinomas [3]. PTX is currently only available in intravenous solutions but, unfortunately, it suffers from poor bioavailability [4] due to its low solubility and permeability in the biological environment [5]. The use of P-gp/P450 inhibitors is a method for increasing PTX bioavailability; however, this strategy often results in severe side effects due to immune response. In order to boost oral PTX solubility and decrease side effects, drug carrier frameworks are being extensively investigated [6–10]. Additionally, bioinformatics can help to better understanding the interactions of drugs and nanocarrier [11, 12].

Nanomedicine is the field of science that deals with organic applications of medicine at the nano-scale level [13–17]. It primarily addresses finding, anticipating, and treating sickness, as well as using nanotechnology to assist in controlling human frameworks at the cellular level [18–25]. For example, niosomes and liposomes have a similar shape, but instead of phospholipids, nonionic surfactants are used for noisome preparation. Niosomes have been proposed as a safer vesicular system than liposomes, with strong chemical consistency [26, 27]. Niosomal vesicles are spherical structures formed by one or more surfactant bilayers with an aqueous phase in between and an adjuvant lipid (cholesterol and ergosterol). Our previous studies showed that using ergosterol instead of cholesterol could improve the physicochemical properties of niosomal formulations. Researchers are working on niosomes because of their apparent advantages, such as cheap price, high stability, and accessibility of surfactants [28]. Niosomes have been suggested as drug delivery systems for anticancer drugs like methotrexate [29], gadobenate dimeglumine [30], doxorubicin, vincristine [31], plumbagin [32], camphtothecin [33], and cisplatin [34]. Besides, there are several studies in which niosomes are used to improve the oral bioavailability of active agents (e.g. phytochemicals, extracts, and drugs) with poor bioavailability. Also, Mo et al. prepared p-phosphonated calix(4)arene vesicles (PCVs) loaded with paclitaxel (PTX) and conjugated with folic acid as a modular drug delivery platform [35].

Despite the significant progress in overcoming many of the original obstacles for effective delivery of anticancer agents, still, there are some limitations for better delivery of drugs. To solve these problems and improve drug levels in selected target tissues or organs, various triggered release approaches based on vesicles have been created. Multiple pH-sensitive systems include pH-responsive peptides or proteins to induce membrane disruption/fusion at lower pH levels [36–39]. Acidic pH in the extracellular environment is one of the characteristic features of diseased tissues (e.g. malignant cells, infection, ischemia, atherosclerosis, and arthritis). The idea of using pH-sensitive vesicles has been inspired by Nature: in fact, it was observed that some viruses enveloped in pathological tissues evolved strategies to take advantage of the acidification of the endosomal lumen to infect cells [40, 41]. Recent research has focused on developing vesicles that are intact under normal circumstances but may be disrupted, and thus, release their components in the slightly acidic environment of tumor tissues. This helps the drug to be released more effectively and selectively from pH-sensitive vesicles into target sites, thereby also reducing side effects [42, 43]. Different types of pH-sensitive vesicles have been suggested in the literature and, among them, cholesteryl hemisuccinate (CHEMS) is the most widely used pH-responsive agent [44]. Furthermore, pH-responsive nanovesicles made of p-phosphonated calix(4)arene have been proposed for cancer therapy [35].

In the current study, we developed a pH-responsive niosome loaded with PTX and investigated its biological efficacy. Span 60, Tween 60, ergosterol, and CHEMS are used as the precursor of niosomes. DLS, optical microscopy, TEM, EE%, release rate in pHs 5.2 and 7.4 were used to evaluate the formulation. Finally, comprehensive in vitro and in vivo assays were performed for assessing the anticancer ability of the developed formulation.

## Materials and methods

### Chemicals and cell lines

Cholesterol hemisuccinate (CHEMS) was obtained from Avanti polar, USA. Paclitaxel (PTX), Span 60, tween 60, and ergosterol were supplied from Sigma-Aldrich Company (USA). Trypan blue, dimethyl sulfoxide (DMSO), 3-(4, 5-dimethylthiazol-2-yl)-2, 5-diphenyltetrazolium bromide (MTT), phosphate-buffered saline (PBS), streptomycin, and penicillin were purchased from Sigma-Aldrich (Steinheim am Albuch, Germany and Sigma-Aldrich, St Louis, MO). Modified Eagle’s medium (DMEM), 0.25% trypsin, and fetal bovine serum (FBS) were supplied by Gibco (Grand Island, NY, USA). All chemicals were of analytical grade. Human breast cancer cell line MCF7, human cervical cancer cell line Hela, and human umbilical vein endothelial cells (HUVECs) were purchased from the cell repository of Pasteur Institute of Iran (Tehran, Iran).

### Synthesis of PTX-loaded niosomes

Niosomes were prepared using the thin-film method. Briefly, Tween 60, Span 60, ergosterol, and CHEMS were mixed at the molar concentrations of 300 µM (0.3: 0.3: 0.3: 0.1 molar ratio) in chloroform. PTX solution (120 ppm) was added to the obtained organic solution. The organic solvents of the solution were removed by Rotavapor (Laboroa 4003, Heidolph, Germany) at 60 °C, 60 min, 180 rpm, and vacuum conditions. At 60 °C, a thin lipid film was formed on the inner surface of a round-bottomed flask and hydrated with ultrapure water. After separating unentrapped PTX by ultracentrifugation at 150,000 × g for 2 h at 4 °C (5415D, Eppendorf, Germany), the PTX-loaded niosome pellet was rehydrated in a certain amount of double-distilled (DD) water. Then, to produce a homogenous emulsion of niosomes, the loaded niosomes were sequentially filtered with 0.45 and 0.22 μm filters (Sartorius AG, Göttingen, Germany). The niosomes without pH-responsive properties were prepared as mentioned above but without using CHEMS. The obtained niosome suspension was kept at 5 ± 3 °C.

### Characterization of PTX-loaded niosomes

#### Size of niosome formulation

DLS characterization of PTX/niosome was carried out using a dynamic light scattering (DLS) (Malvern, Helix, UK) system coupled with a diode-pumped solid-state laser to supply polarized incident light. A digital correlator with a sample range of 25 ns to 100 ms was also included in the device. DLS was conducted by calibrating the intensity scale with toluene against scattering at an angle of 90° to the incident ray. The sample solutions were filtered directly into scattering cells using Millipore Millex filters (0.22 m porosity) and equilibrated for 10 min at the appropriate temperature before being measured. The sampling time was 5–10 min to obtain a fitted correlation function. All of the tests were repeated three times.

#### Morphology and size of niosome formulation

A drop of niosome was mounted on a coverslip and observed using an optical microscope to visualize the appearance of vesicles before filtration (ICC50 W, Germany). Transmission electron microscopy (TEM) was also used to analyze the successful forming of vesicles after filtration (EM10C, Zeiss, Germany). A drop of the dispersion was injected right onto a formvar membrane-coated grid and coated with a drop of 2% (w/w) sodium phosphotungstate solution for TEM examination. Moreover, a filter paper was used to eliminate the residual solution, and the specimens were left to dry in the air.

#### Entrapment efficiency of niosome

Spectroscopic measurements were used to assess entrapment effectiveness. A UV spectrophotometer (Agilent Technologies, Cary 50, USA) was used to determine the concentration of niosomal-encapsulated PTX. The encapsulation efficiency was calculated by the following equation:

$${{{{{\mathrm{Encapsulation}}}}}}\,{{{{{\mathrm{efficiency}}}}}}\left( \% \right) = \frac{{{{{{{\mathrm{PTX}}}}}}\,{{{{{\mathrm{encapsulated}}}}}}}}{{{{{{{\mathrm{Total}}}}}}\,{{{{{\mathrm{amount}}}}}}\,{{{{{\mathrm{of}}}}}}\,{{{{{\mathrm{PTX}}}}}}}} \times 100$$

#### Release study and model of release

A closed dialysis bag (Spectra/Por®, cut-off: 12–14 kDa) containing 1 ml of each formulation was placed in a beaker holding 100 mL of PBS buffers with different pH levels of 5.2, and 7.4. At 37 °C, the buffer was stirred continuously at 150 rpm. 1 mL of the solution was withdrawn at predetermined intervals and replaced with a fresh buffer. The release percentage reflects the percentages of the released drug in the buffer medium. The ratio of the released quantity of formulations in a dialysis bag to the total drug was used to measure these percentages by using UV measurements.

The release kinetics of PTX from the niosome in different pH were evaluated using different kinetic models such as zero-order, first-order, Higuchi, and Korsmeyer-Peppas models based on our previous studies [45]. In this regard, the profile of PTX release (%) against time for the zero-order, the profile of log of the release (%) against time for the first-order, the profile of the release (%) against the square root of time for the Higuchi, and the profile of log of the release (%) against the log of time for Korsmeyer-Peppas models were plotted, respectively, according to the following equations:

$$\begin{array}{*{20}{l}} {{{{{{\mathrm{Q}}}}}}_{{{{{\mathrm{t}}}}}}} \hfill & = \hfill & {{{{{{\mathrm{Q}}}}}}_0 + {{{{{\mathrm{k}}}}}}_0{{{{{\mathrm{t}}}}}}} \hfill \\ {{{{{{\mathrm{Q}}}}}}_{{{{{\mathrm{t}}}}}}} \hfill & = \hfill & {{{{{{\mathrm{log}}}}}}\,{{{{{\mathrm{Q}}}}}}_0 + {{{{{\mathrm{k}}}}}}_1{{{{{\mathrm{t}}}}}}/{{{{{\mathrm{log}}}}}}\,2.303} \hfill \\ {{{{{{\mathrm{Q}}}}}}_{{{{{\mathrm{t}}}}}}} \hfill & = \hfill & {{{{{{\mathrm{k}}}}}}_{{{{{\mathrm{H}}}}}}{{{{{\mathrm{t}}}}}}^{1/2}} \hfill \\ {{{{{{\mathrm{M}}}}}}_{{{{{\mathrm{t}}}}}}/{{{{{\mathrm{M}}}}}}} \hfill & = \hfill & {{{{{{\mathrm{k}}}}}}_{{{{{\mathrm{p}}}}}}{{{{{\mathrm{t}}}}}}^{{{{{\mathrm{n}}}}}}} \hfill \end{array}$$

where Q_t_ is the cumulative percent of drug released at time t, M_t_/M is the fraction of drug released and k_0_, k_1_, k_H_ and k_p_ are the constants for zero-order, first-order, Higuchi and Peppas models. Q_0_ is the total concentration of the loaded drug and n is the exponent related to the release mechanism, which is termed as the diffusional exponent [45].

### Cell culture conditions and viability assay

Cells were grown in medium containing 90% DMEM, 10% heat-deactivated FBS, penicillin (100 U/mL), streptomycin (100 µg/mL), and kept under standard conditions (37 °C, 5% CO_2_). After reaching 80% confluency, cells were harvested and passaged to 75 cm^2^ flasks at the density of 1 × 10^6^ cells/flask.

MTT assay was used to determine the cytotoxic effects of the free and niosome-encapsulated PTX. Cells were plated in 6-well microplates at the density of 5 × 10^3^ cells/well and incubated for 24 h before treatment. Then, cells were exposed to escalating concentrations of free PTX and niosomal PTX (0.1, 0.2, 0.4, 0.8, 1.6, and 3.2 µg/mL). Untreated cells were considered as controls. Following 48 h incubation, 200 µL of the MTT reagent (5 mg/mL diluted in PBS) was added into each well and kept in an incubator at 37 °C for 3 h. After discarding the culture media, samples were dissolved in 200 µL DMSO and transferred to another 96-well microplate. The absorbance of dissolved formazan crystals was read at 570 nm using a SpectraMax microplate reader (Molecular Devices, Sunnyvale, CA). A total of three independent experiments were conducted for this assay. Results are presented as the percentage of viable cells calculated via dividing the absorbance measured for treated cells by the absorbance measured for control (non-treated) cells. The IC50 for free drug and encapsulated drug was estimated by GraphPad prism software version 6.01 (GraphPad software Inc., San Diego, California. USA).

### Morphological alterations

Cells (2 × 10^5^ cells/well) were seeded in 12-well plates and treated with different concentrations of free and nano-encapsulated PTX for 48 h. Cells were photographed under phase-contrast microscopy and imaged using a digital camera.

### Animal treatments and experimental design

Forty male adult Sprague-Dawley rats (mean eight 228 g) were purchased from the laboratory animal center of the University of Zabol, Zabol, Iran. Rats were housed under standard room temperature (23 °C) and natural light/dark cycles in a well-ventilated room. Animals had free access to tap water and a rodent diet during the procedure of the experiment. The injections and rat handling were performed according to the guidelines of care and the use of laboratory rodents, NIH publication no. 85–23. Before the injections, animals were divided into four equal groups: the control group was treated with physiological saline intraperitoneally for two weeks, the animals in the second group were administered with niosomal PTX (2.5 mg/kg BW), and the other two groups received niosomal PTX (5 mg/kg) and free PTX intraperitoneally for fourteen days, respectively. The dose was selected based on our preliminary experiments and previous studies. Finally, blood samples were collected from the heart. The collected blood was divided into two samples: one sample was centrifuged (3000 rpm for ten min) to separate the serum, while the second sample was sent to the clinical pathology laboratory to determine some hematological indexes such as hematocrit (HCT), hemoglobin concentration (HB), and red blood cell count (RBC count).

### Serum biochemical parameters

The serum levels of blood urea nitrogen (BUN) and creatinine were measured according to Pars Azmoon reagent kits instructions (Pars Azmoon. Co., Tehran, Iran). All biochemical analyses were performed using the Selectra Pro M autoanalyzer, (Vital Scientific, Netherlands). A colorimetric assay was used to determine serum aspartate aminotransferase (AST) and alanine aminotransferase (ALT) levels. Serum AST and ALT levels were measured by using Pars Azmoon reagent kits (Pars Azmoon, Tehran, Iran).

### Malondialdehyde (MDA) values

Liver malondialdehyde contents, a measure of liver lipid peroxidation, were measured using the Ohkawa method [46].

### Histopathological examination

After two-week intraperitoneal administration, rats were euthanized by 1.5% pentobarbital sodium (200 mg/kg) followed by cervical dislocation. Liver and kidney specimens were sliced and preserved in 10% neutral buffered formalin for two days to ensure complete tissue fixation. After paraffin embedding and cutting on a rotary microtome, paraffin blocks were cut into 5 μm-thick micro-slides. The histopathological sections were stained with hematoxylin–eosin and examined under a light microscope (Tokyo, Olympus, Japan). For semi-quantitative analysis, the hepatic and renal lesions were graded from zero (normal histology) to three (severe pathological lesions), as shown in Table 1.

**Table 1:** The classification of hematoxylin and eosin-stained sections injuries - hepatic damage score (HDS), renal damage score (RDS)

Description		Degree			
		0	1	2	3
H&E	Liver	Normal	Normal hepatocytes with slight sinusoidal disarrangement	Sever fatty change. Severe sinusoidal disarrangement.	Hepatocyte necrosis
	Kidney	Normal	Mild narrowing of proximal and distal tubules. Mild expansion of Bowman’s capsule	Vacuolation of proximal and distal tubules.	Glomerulosclerosis

### Statistical analysis

The SPSS software (version 20.0) was used to analyze the biochemical and histopathological. Statistical analyses were performed by using the one-way analysis of variance (ANOVA). The Tukey posthoc test was used to investigate the statistical difference between groups. The statistically significant level was set at *P* < 0.05.

## Results

### Physicochemical characterization of PTX-loaded niosomes

**Fig. 1:**
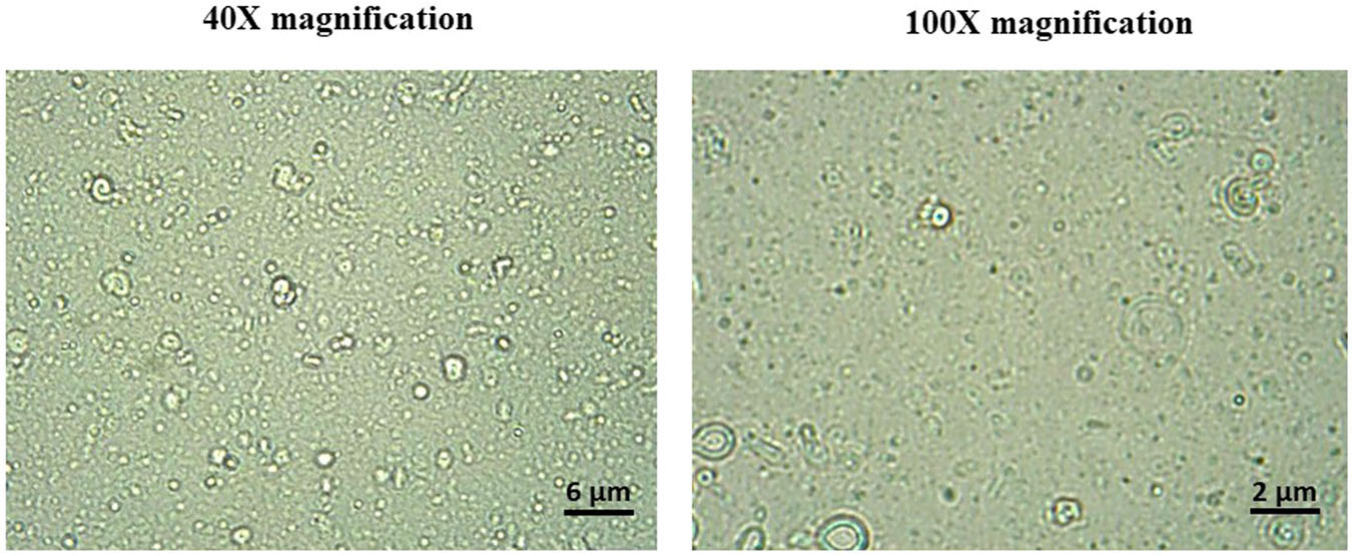
Optical microscopic images of pH-responsive PTX/niosome before niosome filtration at the magnifications of 40× and 100×

The pH-responsive vesicles were prepared using a conventional chloroform film method, generally used to prepare niosomes. Based on our previous experiences and also other studies, this niosome preparation method was found to be repeatable in terms of size distribution and percentage of drug loading. The morphology of the niosome formulation before the filtration procedure can be seen in the photomicrographs taken with an optical microscope and displayed in Fig. 1. In the micrographs, spherical and multi-layer vesicles (MLVs) were frequently seen.

**Fig. 2:**
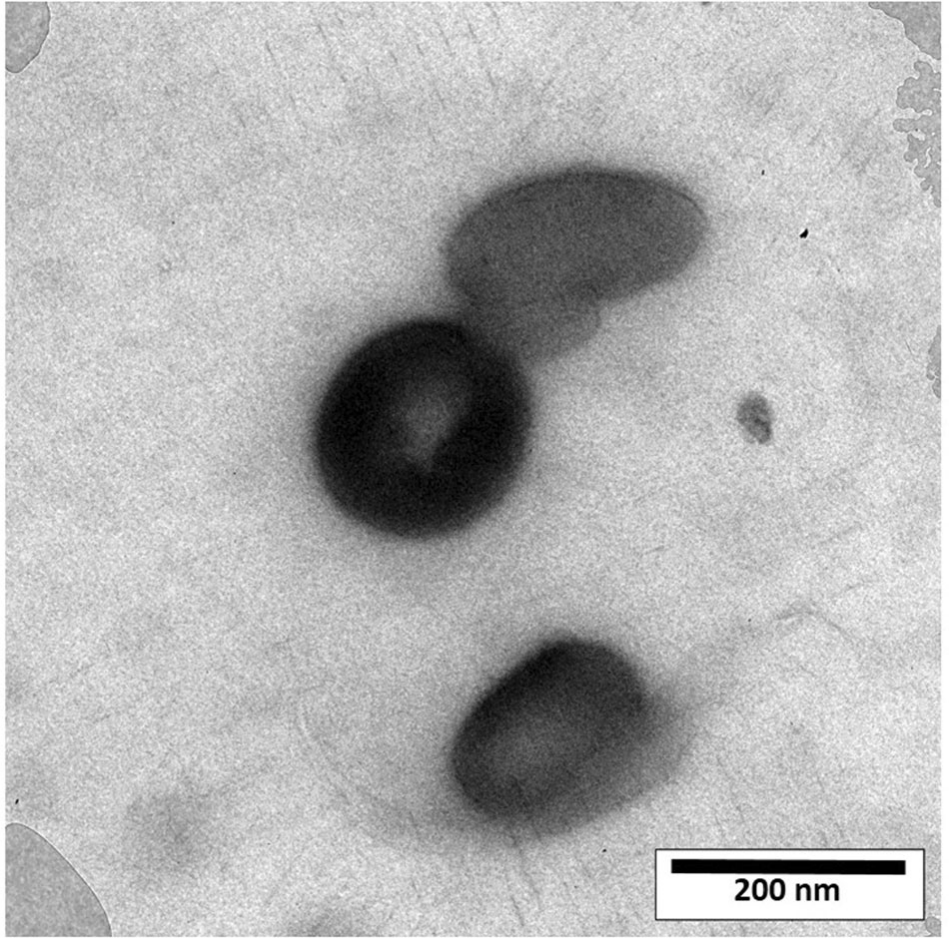
TEM image of pH responsive PTX/niosome (pH = 7.4), original magnification: 40,000×

The shape, size, and morphology of niosomes after filtration were observed by TEM, a reported in Fig. 2. The vesicles were monodispersed, and the formulation had an approximately spherical shape without aggregation. Furthermore, TEM image revealed that niosomes are hollow vesicular structures. On the other hand, there was no structural deformation for our formulation, suggesting the high stability of the prepared niosomes.

**Fig. 3:**
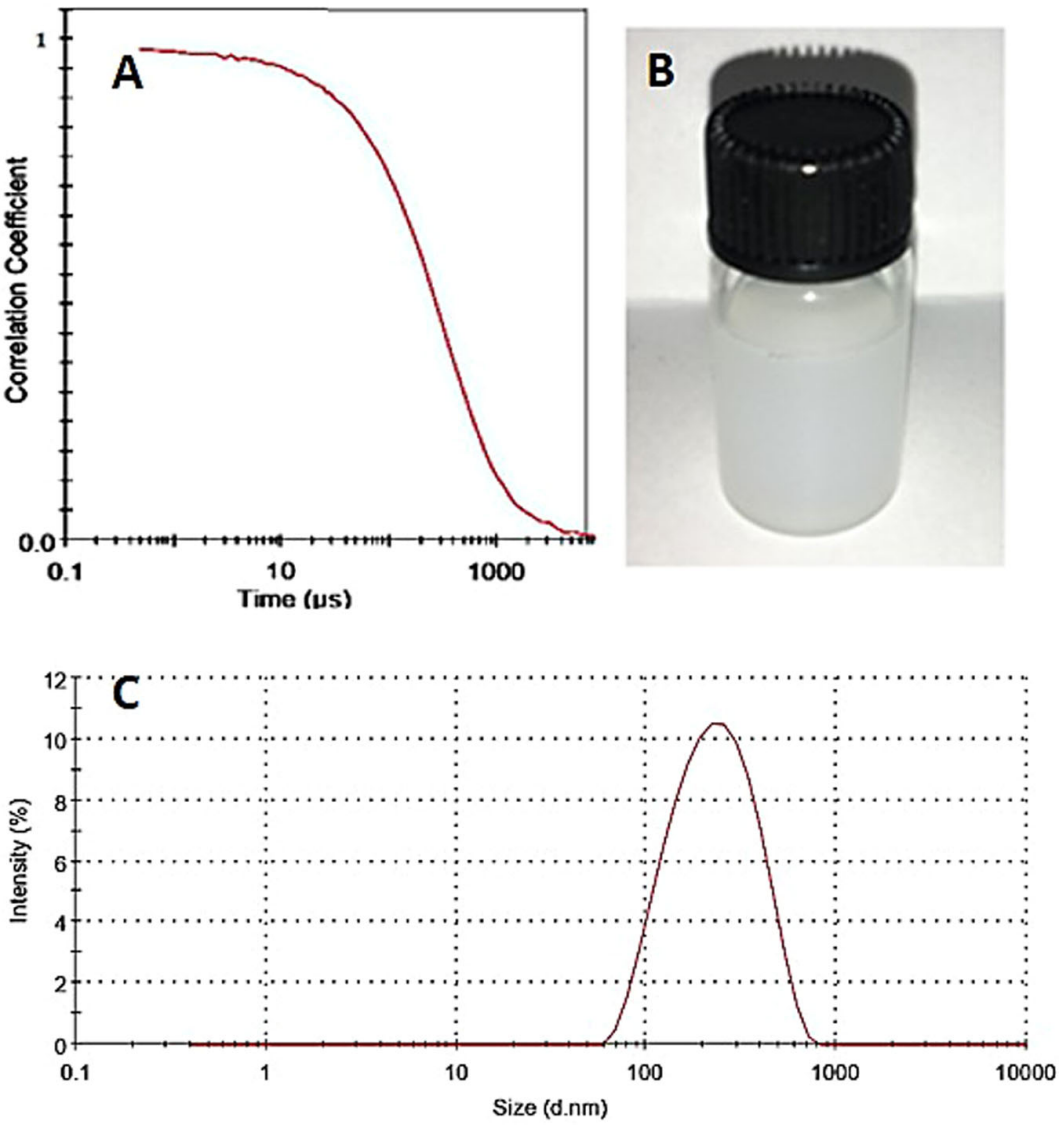
**A** Correlation function of pH-responsive PTX/niosome (**B**) stability of formulation after three months and (**C**) size distribution of niosomes measured by DLS

Figure 3A shows the correlation function of pH-responsive PTX/niosome assessed by DLS analysis. The single exponential function of the autocorrelation function of samples provided by the DLS confirms the monodispersity of niosomes. Additionally, after niosome storage at 4 °C for over three months, the homogeneity and stability of the formulation were retained, thus revealing high storage stability (Fig. 3B). As seen in Fig. 3B, the macroscopic appearance of the formulation was a white to slightly yellow homogeneous and translucent suspension. Figure 3C shows the size distribution of PTX/niosome with an average size of 240 nm. In this regard, the size of the niosomes is sufficient for tumor-specific accumulation [47]. These findings appear to be in line with those obtained via the TEM measurement.

### Entrapment efficiency

Encapsulation efficiency (EE) is one of the most critical physico-chemical parameters in niosome formulations. According to previous researches, niosomes formulated using the thin film technique had a higher EE than those prepared by other techniques [48]. The drug bioavailability is ensured by the high encapsulation performance, and the concentration of PTX in the vesicle will be increased at higher EE. EE for PTX was 77.0 ± 2.3 %, indicating the successful loading of PTX into niosomes.

### In vitro release experiment

**Fig. 4:**
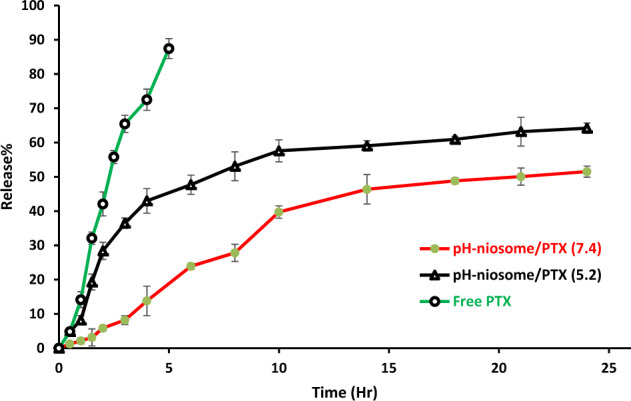
Release (%) of PTX from pH-responsive PTX/niosome in PBS solution with different pH levels of 5.2, and 7.4 at 37 °C. Points, mean (*n* = 3); bars, SD

Figure 4 depicts the release of PTX from the prepared niosomal formulations and reveals that the release behavior is dependent on pH. The drug release rate is an important factor in drug delivery systems that requires deeper investigations. Incubation in buffer was used to analyze drug release from niosomes, and the formulations were evaluated by monitoring the absorbance shifts of the withdrawn samples. The curves illustrate the in vitro release of free PTX (pH = 7.4) and pH-responsive PTX/niosomes at pH 5.2 and 7.4 (Fig. 4). In both loaded formulations, a double-stage release of PTX was observed, with an initial relatively rapid drug release followed by an equilibrium plateau or a slower release period. After 4 hours, 90 % of the free PTX was released (pH = 7.4), while a remarkably controlled release of PTX from niosomal formulations was measured for up to several hours, according to the release profile. The resulting release data showed a significant dependence of the drug on pH conditions (*p* < 0.05). By changing the pH values, the release rate of the pH-responsive formulations increased due to the presence of CHEMS in the niosomal bilayer.

**Fig. 5:**
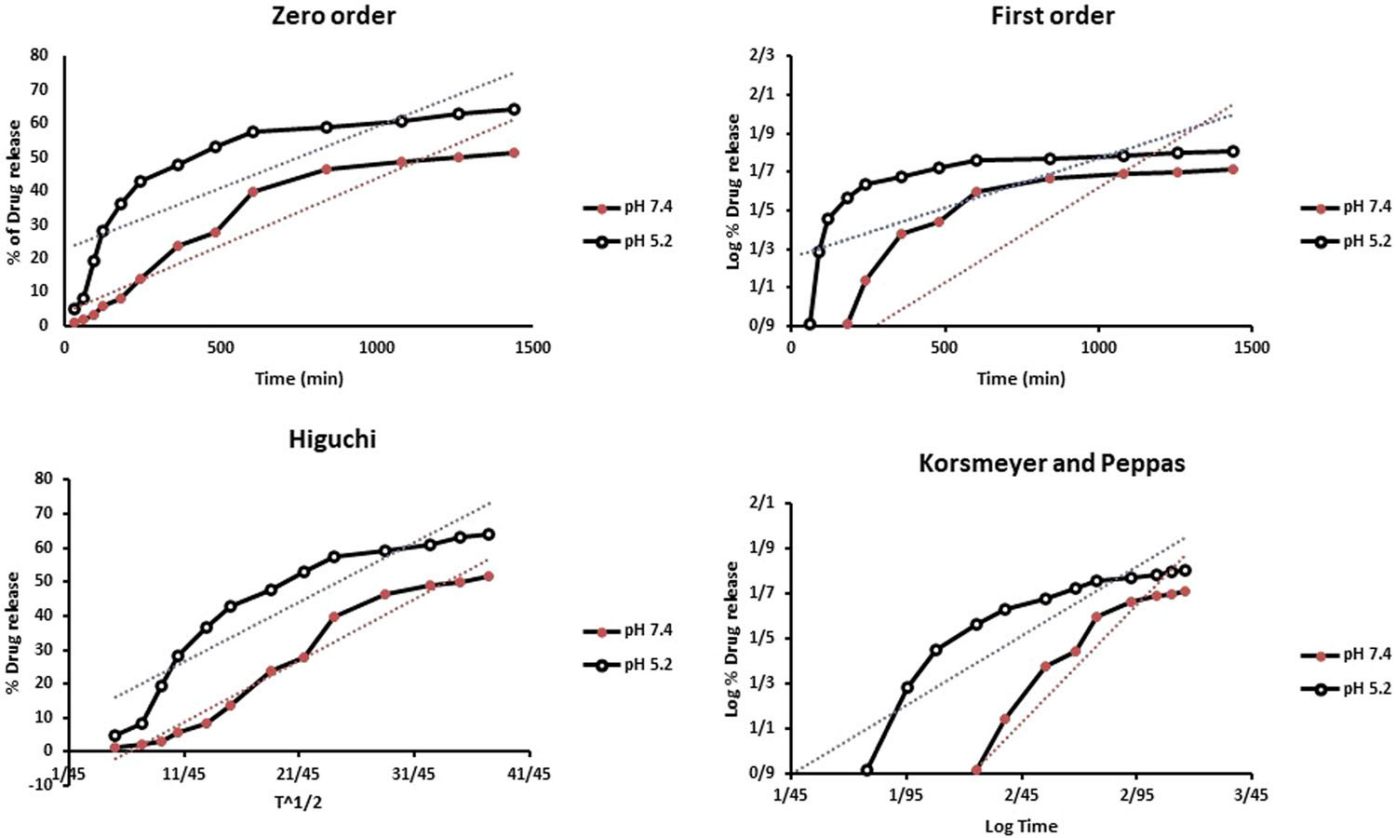
Profiles of different kinetic models for release of PTX from pH-responsive PTX/niosome in PBS solution with different pH levels of 5.2, and 7.4 at 37 °C

To better assess the release kinetics of PTX from niosomal formulations in vitro, data from both buffers (pH 5.2 and 7.4) were fitted by using mathematical models and proper kinetic parameters, as illustrated in Fig. 5. After matching the first-order, zero-order, Higuchi, and Korsmeyer–Peppas models with the PTX release profile, it was found that the kinetics of release for pH 7.4 followed the Korsmeyer–Peppas model with a coefficient R^2^ of 0.97. On the other hand, PTX release at pH 5.2 can be well described by the Higuchi model with a coefficient R^2^ of 0.86. A summary of kinetic models, equations and R^2^ of PTX-loaded pH-responsive niosomes is reported in Table 2.

**Table 2:** Kinetic models (linearized equations) of PTX released from pH-responsive niosomes at pH 7.4 and 5.2

Kinetic model	pH	Equation	R^2^
Zero order	7.4	*y* = 0.0397x + 4.1231	0.9064
	5.2	*y* = 0.0362x + 23.109	0.7084
First order	7.4	*y* = 0.001x + 0.6344	0.6982
	5.2	*y* = 0.0005x + 1.2596	0.4799
Higuchi	7.4	*y* = 1.8111x − 11.861	0.9663
	5.2	*y* = 1.7636x + 6.281	0.8596
Korsmeyer and Peppas	7.4	*y* = 1.0488x − 1.4461	0.9716
	5.2	*y* = 0.617x + 0.0015	0.8489

### Cytotoxic and morphological assessments

**Fig. 6:**
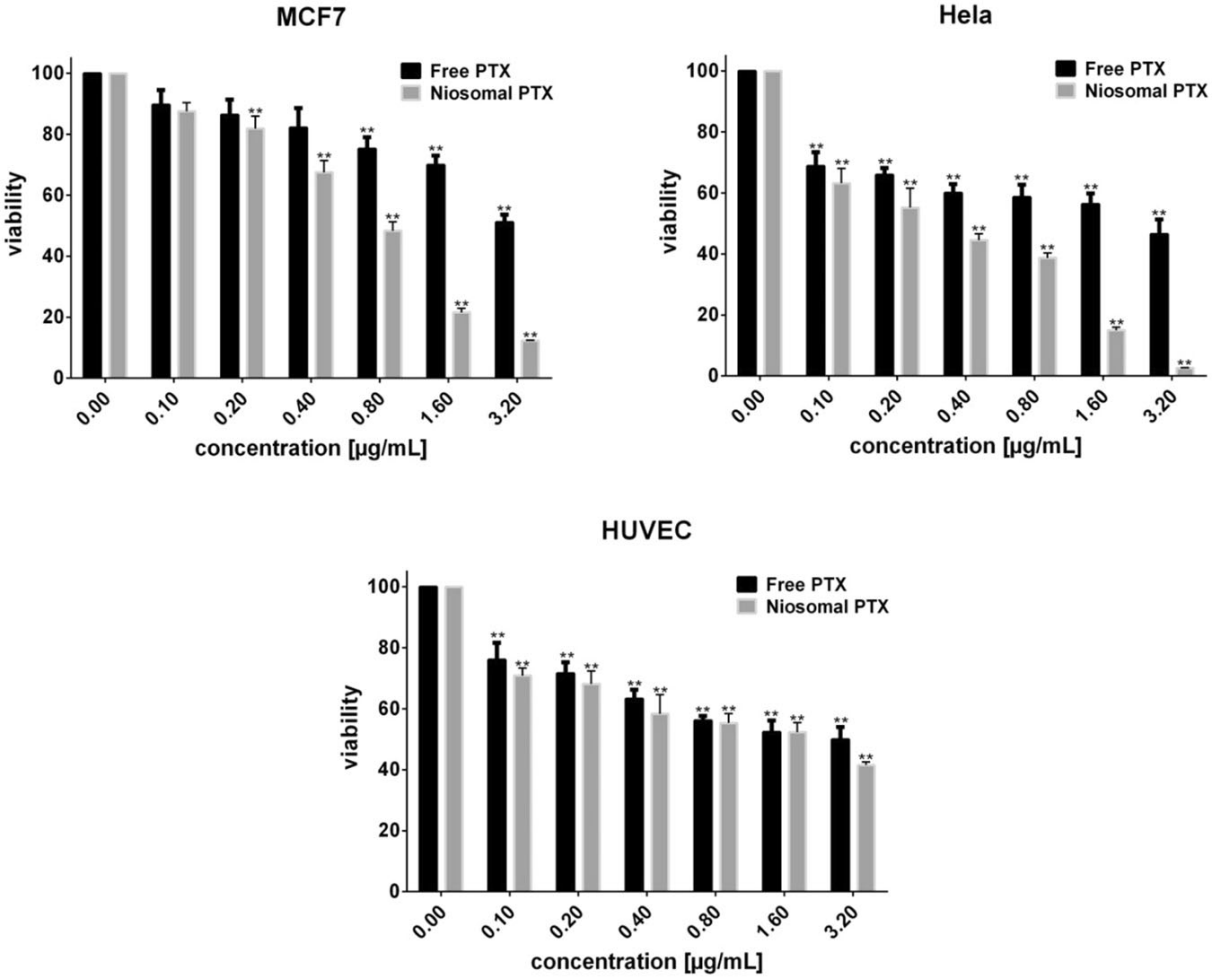
Cytotoxicity evaluation of free PTX and niosomal PTX on MCF7, Hela, and HUVEC cells after 48 h exposure. (**P < 0.05 compared with untreated cells)

Figure 6 shows the viability of the studied cell lines upon 48 h exposure to free PTX and niosomal PTX as a function of concentration. Compared with control cells, free PTX significantly diminished the viability of Hela, MCF7, and HUVEC cells following a concentration-dependent fashion (*P* < 0.05). IC50 values corresponding to the exposure of MCF7, Hela, and HUVEC cells to free PTX were 2.973, 1.194, and 1.268 μg/mL, respectively, while these values decreased to 0.681 μg/mL (for MCF7 cells), 0.281 μg/mL (for Hela cells), and 1.001 μg/mL (for HUVEC cells) when treated with increasing concentrations of niosomal PTX. We observed that Hela cells were the most sensitive to free- and niosomal PTX.

**Fig. 7:**
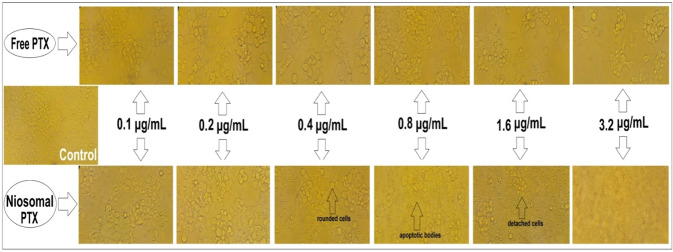
Hela cells exposed to 0 to 3.2 µg/mL of free PTX or niosomal PTX for 48 h. Morphological changes were monitored by invert microscopy

**Fig. 8:**
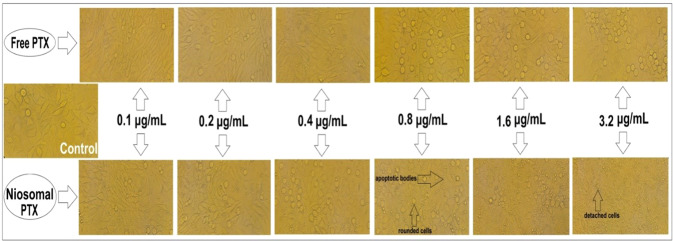
MCF7 cells treated with 0 to 3.2 µg/mL of free PTX or niosomal PTX for 48 h. Morphological alterations were observed by invert microscopy

Monitoring of cell morphology revealed that treatment with free PTX (0.1–3.2 μg/mL) only decreased the number of viable Hela (Fig. 7) and MCF7 (Fig. 8) cells, while their morphology did not undergo significant alterations. On the contrary, niosomal PTX induced concentration-dependent morphological changes on Hela (Fig. 7) and MCF7 (Fig. 8) cell lines. In this regard, exposing cells to high concentrations of niosomal PTX (≥0.4 μg/mL in Hela cells and ≥0.8 μg/mL in MCF7 cells) significantly diminished the number of viable cells, induced progressive nuclear shrinkage, and caused the formation of apoptotic bodies. When cancer cells were treated with high concentrations of niosomal PTX (>1.6 μg/mL) for 48 h, cells became separated, rounded, and detached from the culture dish. These morphological alterations were not observed when cells were exposed to free PTX at given concentrations.

### Biochemical results

As shown in Table 3, the rats treated with free PTX had higher concentrations of serum BUN and creatinine, which were statistically significant compared to the control group (*P* < 0.01). Serum AST and ALT levels were also higher in the rats treated with free PTX as compared to the controls (*P* < 0.001) (Table 3). In addition, the rats which were treated with free PTX had higher liver MDA levels when compared to the healthy control group (*P* < 0.01). There was no significant difference in haematocrit and hemoglobin concentration and red blood cell count in rats receiving free PTX compared to the normal control rats (*P* > 0.05).

**Table 3:** Effects of free PTX and niosomal PTX on hematological parameters, biochemical parameters, and liver MDA content of different experimental groups

Parameter	Treatment*			
	Control	Niosomal PTX 2.5 mg/kg	Niosomal PTX 5 mg/kg	Free PTX 2.5 mg/kg
MDA(nmol/mg protein)	201.7 ± 46.4	222.8 ± 27.4	245.7* ± 25.5	256.2** ± 34.5
AST (U/L)	148.8 ± 24.7	153.7 ± 31.5	175.3* ± 15.1	197.6*** ± 13.4
ALT (U/L)	38.8 ± 5.1	39.4 ± 5.0	49.6* ± 9.7	54.7*** ± 10.1
BUN (mg/dL)	14.1 ± 2.9	18.9* ± 3.8	20.4** ± 4.4	20.7** ± 4.2
Creatinine (mg/dL)	0.82 ± 0.12	0.91 ± 0.11	1.10* ± 0.20	1.20** ± 0.34
Hemoglobin (g/dl)	13.9 ± 1.4	14.6 ± 1.1	13.7 ± 2.1	14.7 ± 4.2
Haematocrit (%)	38.8 ± 8.4	41.0 ± 3.5	43.3 ± 6.3	40.2 ± 5.1
RBC 10^6^ Cells/mm^3^	6.7 ± 0.2	6.8 ± 0.21	6.6 ± 0.4	6.5 ± 0.6

*PTX* paclitaxel, *AST* aspartate aminotrasferase, *ALT* alanine aminotransferase, *BUN* blood urea nitrogen

*significant for control group (*P* < 0.05)

**significant for control group (*P* < 0.01)

***significant for control group (*P* < 0.001)

Treatment with niosomal PTX at the dose of 5 mg/kg also increased ALT and AST levels compared to the normal control rats. The statistical analysis showed a significant difference in serum BUN and serum creatinine levels of rats treated with a 5 mg/kg dose of niosomal PTX compared to those in healthy rats (*P* < 0.01 and *P* < 0.05, respectively). On the other hand, there was no significant difference in hematocrit, red blood cell count and hemoglobin concentration in rats treated with niosomal PTX (5 mg/kg) compared to the control rats (*P* > 0.05). There was also no significant difference in serum AST, ALT, BUN, and creatinine levels of tats treated with niosomal PTX (2.5 mg/kg) compared to the normal control rats (*P* > 0.05). Liver MDA levels did not also significantly change in rats receiving the 2.5 mg/kg dose of niosomal PTX compared to the normal control rats. In contrast, liver MDA levels of rats receiving the 2.5 mg/kg and 5 mg/kg of niosomal PTX were significantly higher than the normal control rats.

### Histopathological investigation

Table 4 shows the hepatic damage score (HDS) in hematoxylin eosin-stained sections of different groups.

**Table 4:** The hepatic damage score (HDS) in hematoxylin eosin-stained sections of different groups

	H&E	
	Liver	Kidney
Control	0.30 ± 0.48	0.60 ± 0.51
Niosomal PTX 2.5 mg/kg	0.50 ± 0.70	0.80 ± 0.63
Niosomal PTX 5 mg/kg	1.40* ± 0.70	1.4 ± 1.0
Free PTX 2.5 mg/kg	1.8** ± 0.9	1.9** ± 0.7

*significant concerning control group (*P* < 0.05)

**significant concerning control group (*P* < 0.01)

**Fig. 9:**
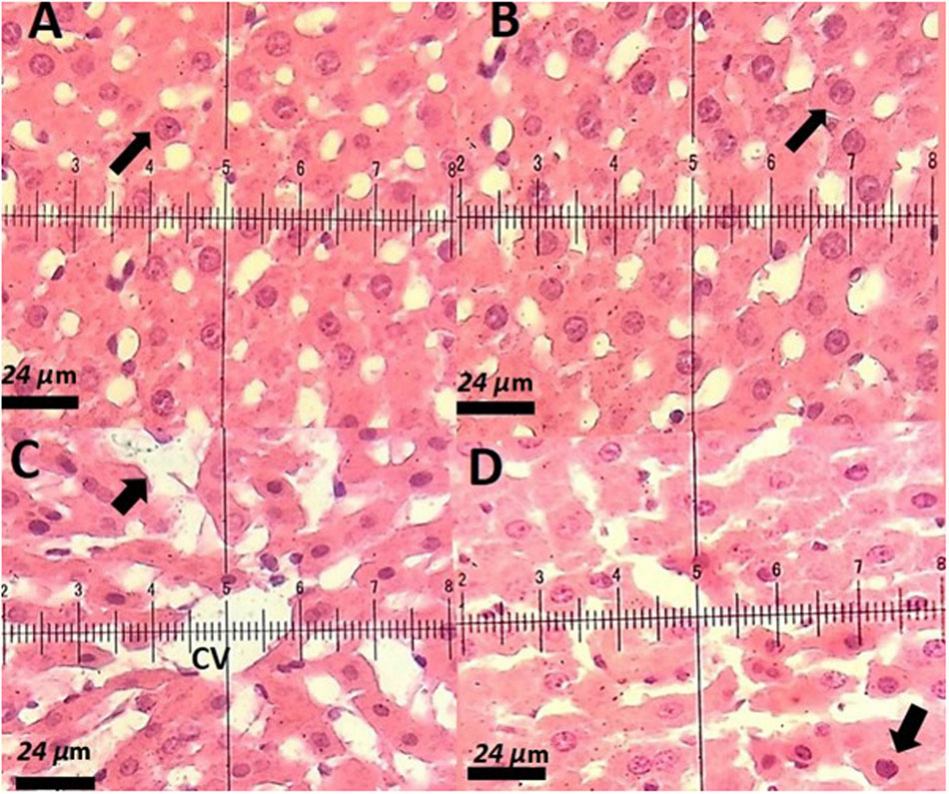
**A** Hematoxylin-eosin-stained section of a control rat showing characteristic normal liver histopathology. The arrow indicates a normal hepatocyte with a normal nucleus and cytoplasm. H&E staining (×40); **B** Liver sections of rat vacuolation (arrow).H&E staining (×40); **C** Liver section of a PTX-treated rat, a decrease in cytoplasmic vacuolization, arrow point indicates the hepatocytes regeneration. H&E staining (×40); **D** Liver section of a rat treated with niosomal PTX, a decrease in pyknotic nuclei, H&E staining (×40)

**Fig. 10:**
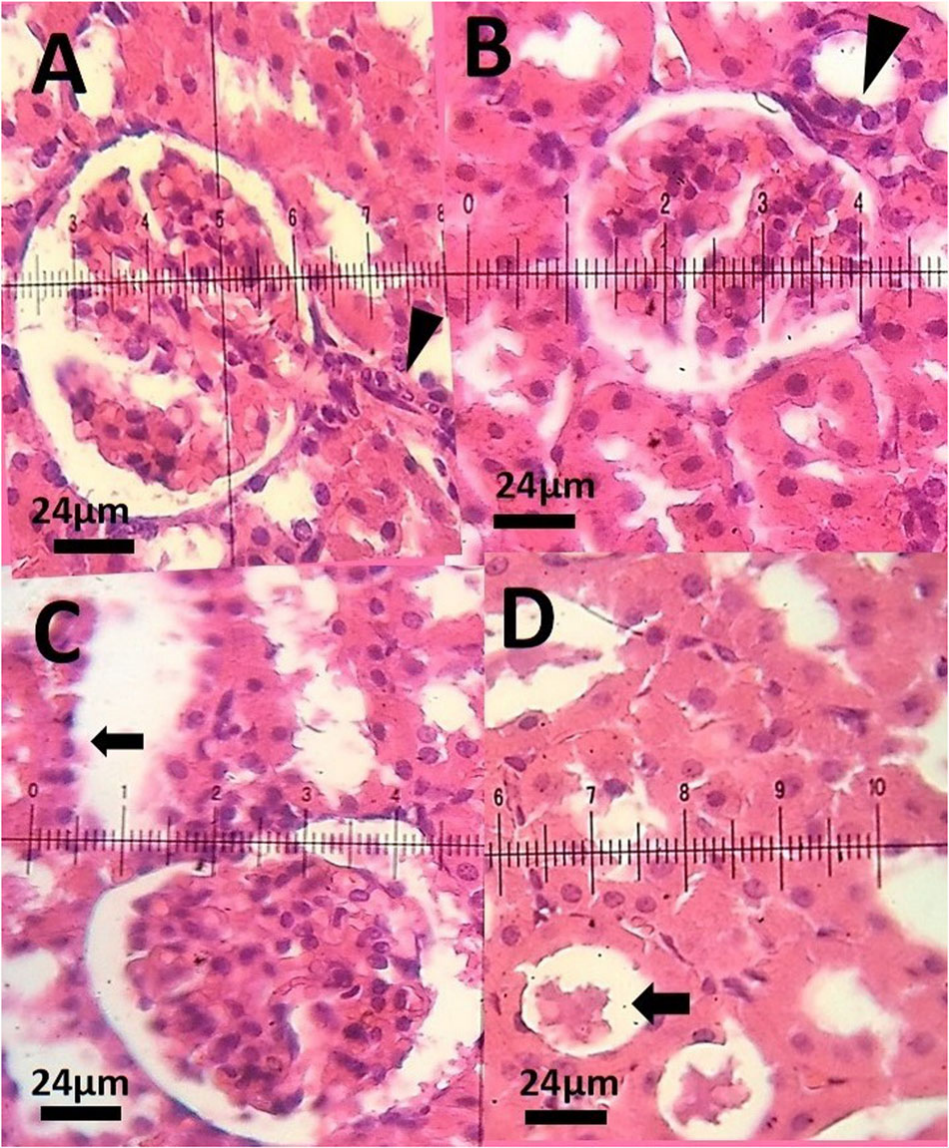
**A** Hematoxylin–eosin-stained kidney sections of a control rat showing (G) normal glomeruli, macula densa apparatus, and normal proximal tubules H&E staining (×40); **B** Kidney sections of a rat received the 2.5 mg/kg dose of niosomal PTX showing normal glomerulus and normal macula densa (arrow) H&E staining (×40); **C** Kidney section of a rat received the 5 mg/kg dose of niosomal PTX, arrow shows cellular vacuolation of renal tubules. H&E staining (×40); **D** Kidney section of a rat treated with free PTX, narrowing of proximal tubules and hyaline casts (arrow), H&E staining (×40)

In the histopathological investigations, control rats had normal hepatocytes structure, normal sinusoids, well-arranged hepatic lobule, and well-distinct central vein in each hepatic lobule (Fig. 9A). The microscopic investigation (hematoxylin & eosin-staining) of the liver of rats receiving the 2.5 mg/kg dose of niosomal PTX showed well-arranged sinusoids and liver cells (Fig. 9B). Histopathological analysis of rats receiving the 5 mg/kg dose of niosomal PTX showed sinusoidal disarrangement and nuclear pyknosis (Fig. 9C). These changes were more severe in a liver section of rats receiving the free PTX (2.5 mg/kg). The histological analysis of the liver of rats treated with free PTX showed severe necrosis and fatty change (Fig. 9D). These changes were less severe in rats treated with niosomal PTX (Fig. 9C). The histopathological analysis of kidney sections showed normal renal structure in control rats and rats receiving the 2.5 mg/kg dose of niosomal PTX. As shown in Fig. 10A, control rats had normal glomerular structure, normal proximal tubules, and well-distinct macula densa apparatus near the distal tubules. The histopathological analysis of the kidney of rats receiving the 2.5 mg/kg dose of niosomal PTX showed normal glomeruli, proximal tubules, and macula densa apparatus (Fig. 10B). Histopathological analysis of rats receiving the 5 mg/kg dose of niosomal PTX showed vacuolation of proximal and distal tubules (Fig. 10C). The vacuolation of proximal and distal tubules was more severe in renal sections of rats receiving the free PTX (2.5 mg/kg). The histopathological investigation of the kidney of rats treated with free PTX showed hyaline cast formation and narrowing of proximal and distal tubules (Fig. 10D).

## Discussion

Nowadays, niosomes have gained much attention in drug delivery applications because they can entrap both hydrophobic and hydrophilic drugs and help treat various tumors a well as microbial and viral diseases. In the present study, PTX loaded into the pH-responsive niosome as a new drug delivery system for cancer therapy. Microscopy evaluation showed a multi-layer and spherical morphology for niosomes. Asthana et al., in a similar paper, used optical microscopy to observe the morphology of the prepared clarithromycin loaded-niosomes that were spherical in shape, too [49]. In another study, Bansal et al. developed cefdinir niosomes for oral delivery and also reported a round shape of vesicles [50]. TEM image revealed that niosomes are hollow vesicular structures. These observations are consistent with the results reported by Asthana et al. for clarithromycin niosomes [49] and by Xu et al. for curcumin-loaded niosomes [51]. On the other hand, there was no structural deformation for our formulation, suggesting the high stability of the prepared niosomes. The single exponential function of the autocorrelation function of samples provided by the DLS confirms the monodispersity of niosomes. Additionally, after niosome storage at 4 °C for over 3 months, the homogeneity and stability of the formulation were retained, thus revealing high storage stability. EE for PTX was 77.0 ± 2.3 %, indicating the successful loading of PTX into niosomes. Because of the hydrophobic nature of PTX, it is incorporated into the lipid bilayer of niosomes. Alemi et al. loaded PTX and curcumin in cationic PEGylated niosomal formulations. Their results showed extremely high entrapment efficiency (~ 100% for both therapeutic drugs) [52]. This high EE% was associated to their novel cationic PEGylated niosomal formulations (Tween-60: cholesterol: DOTAP: DSPE-mPEG = 59.5: 25.5: 10: 5)). The resulting release data showed a significant dependence of the drug on pH conditions (*p* < 0.05). By changing the pH values, the release rate of the pH-responsive formulations increased due to the presence of CHEMS in the niosomal bilayer. CHEMS probably reduces the integration of the niosome bilayers as the pH of the region decreases, resulting in turbulence and an increased release rate. Due to acid accumulation, tumor cells have an intracellular pH of about 5.2. Assuming that in vitro results are predictive of what happens in vivo to some extent, a quicker release from niosomes is expected in acidic pH, i.e. in the presence of target cancer cells. These results are consistent with the findings of other studies about drug release from niosomes, which are known to play an important role in developing controlled-release formulations for the delivery of active compounds. In a similar study, Alemi et al. [52] evaluated PTX and curcumin co-administration in cationic PEGylated niosomal formulations. They reported that 29.93 and 28.16 % of curcumin and PTX were released after 72 h, respectively. Curcumin and PTX had a double-stage cumulative release profile as well, with a rapid initial release period accompanied by a slower release period [52]. In another study, Bayindir et al. [53] characterized niosomes prepared with various non-ionic surfactants for PTX oral delivery. PTX release from niosomes followed a diffusion-controlled mechanism and the authors claimed that the slow release observed from these formulations might be beneficial for reducing the toxic side effects of PTX [53]. In another study, Mo et al. evaluated the EE% and release rate of PTX-loaded pH-responsive nanovesicles. Based on the results, EE% was about 90% with a faster release behavior at pH 5.5 as compared to pH 7.4 [35]. It was found that the kinetics of release for pH 7.4 followed the Korsmeyer–Peppas model with a coefficient R^2^ of 0.97. This suggests that PTX release was controlled by swelling. On the other hand, PTX release at pH 5.2 could be well described by the Higuchi model with a coefficient R^2^ of 0.86. This model suggests that the drug release is a diffusion process based on Fick’s law which is square root time-dependent. For cefdinir niosomes, Bansal et al. [50] conducted a kinetic release study and found that the drug release mechanism followed the Higuchi model. Furthermore, the release exponent (n) for all of their formulations was less than 1, suggesting anomalous transport [50]. Suriyaprakash et al. formulated PTX-loaded niosomes by using Span 60 and assessed drug entrapment and cytotoxicity. They found the cumulative release percentage of 95.6% after 17 h, and the LD50 of 9.87 µg/mL for HEP-2 and 6.2 µg/mL for the Hela cell line [54]. Alemi and co-workers examined the cell-killing effects of niosomal PTX, prepared by the thin-film hydration method, on PC3 prostate cancer cells. They found that niosomal PTX prepared with sorbitan monostearate and cholesterol exhibited higher toxicity than free PTX on these cancer cells (IC50: 17.09 vs. 25.4 μg/mL for niosomal PTX and free PTX, respectively) [55]. In agreement with these findings, compared with free PTX, we observed markedly lower IC50s for Hela and MCF7 cells treated with niosomal PTX, suggesting that PTX entrapment might improve its delivery to tumor sites. Other studies also focused on developing niosomal PTX that can be applied in targeted drug delivery. Findings of another study by Ashrafi and colleagues indicated that a combination of PTX with curcumin using a novel niosomal drug delivery system was more effective against MCF7 breast cancer cells than their free forms [52]. Also, Ashrafi and her research team found that PTX-loaded niosomes had high efficacy as adjuvant therapy for clinical usage against Nalm-6 leukemia cells [56]. Finally, Zarei et al. showed that cytotoxicity of poly(ethylene glycol)-based and non-poly(ethylene glycol) niosomes prepared by using Span 60 were significantly higher than free PTX (lower IC50s) [57].

The current work aimed at comparing the sub-acute toxicity of free PTX and niosomal PTX in a rat model. Our results showed that sub-acute treatment with free PTX can increase serum liver enzymes and serum kidney markers in rats. Intraperitoneal injections of free PTX induced histopathological changes in kidney and liver histopathological sections. Results of the current study show that treatment with free PTX can increase hepatic MDA, which is an excellent biomarker of lipid peroxidation. The results indicate sub-acute toxicity of free PTX in rats and are consistent with early evidence of previous experiments showing that treatment with PTX could have toxic effects and induce liver and kidney histopathological changes. To the best of our knowledge, however, there is a paucity of available data in the literature regarding the sub-acute toxicity of niosomal PTX. Our results also indicate the potential nephrotoxicity and hepatotoxicity of niosomal PTX, especially at high doses. Overall, the current study suggests that both free PTX and niosomal PTX can have toxic effects at high doses. It could be concluded that both free PTX and niosomal PTX could have dose-dependent toxic effects on the liver and kidney of rats. Overall, it seems that niosomal PTX has fewer side effects compared to free PTX. The histopathological results show that both the free form and niosomal form of PTX can easily cross biological barriers and accumulate in the liver and kidney.

## Conclusions

In this study, pH-responsive niosomes were loaded with PTX and modified with ergosterol. Results showed that the PTX/niosome formulation has a nanometric size and spherical morphology. EE% of PTX was about 77%, and a pH-dependent release of PTX was observed. Besides, our novel niosomal formulation of PTX showed high efficacy against human cancers derived from cervix and breast tumors. Furthermore, the in vivo experiments demonstrated the fewer toxic effects of niosomal formulation of PTX compared to the free PTX. Overall, our results provide proof of concept for encapsulating PTX in niosomal formulation to enhance its therapeutic efficacy.

## Data Availability

Data are reported in the manuscript.
